# Exploring the Role of Wearable Electronic Medical Devices in Improving Cardiovascular Risk Factors and Outcomes Among Adults: A Systematic Review

**DOI:** 10.7759/cureus.36754

**Published:** 2023-03-27

**Authors:** Kofi Seffah, Mustafa Abrar Zaman, Nimra Awais, Travis Satnarine, Ayesha Haq, Grethel N Hernandez, Safeera Khan

**Affiliations:** 1 Internal Medicine, California Institute of Behavioral Neurosciences & Psychology, Fairfield, USA; 2 Internal Medicine, Piedmont Athens Regional Medical Center, Athens, USA; 3 Research, California Institute of Behavioral Neurosciences & Psychology, Fairfield, USA; 4 Pediatrics, California Institute of Behavioral Neurosciences & Psychology, Fairfield, USA

**Keywords:** awareness, heart disease, modifiable risk factors, motivation, physical activity, predictive tools, prevention, wearable electronic devices

## Abstract

There is a developing trend of using wearable electronic devices as health aides, spurred on by telecommunication companies as fitness devices and marketed as such. They have been shown to count steps, pulse, and record arrhythmias, doubling as communication devices and prompting healthcare providers in some instances. We sought to determine if there was a direct correlation between device use and increased physical activity as recommended by the World Health Organization, or if this physical activity increase was only marginal at best. In addition, we sought to investigate if there were additional benefits to using these devices besides increased self-awareness of health. This systematic review used Preferred Reporting Items for Systematic Reviews and Meta-Analyses guidelines. Keywords for searching articles centered around cardiovascular disease, wearable electronic devices, and their synonyms. Most of the data were obtained from PubMed, although other contributing databases were used, including ResearchGate, Science.gov, ScienceDirect, and PubMed Medical Subject Headings database. Only full-text articles were used. We identified 62 articles that met our search criteria but narrowed them down to 19 following qualitative assessment. Increased physical activity was found to be the one parameter that stood out by way of benefit from the device. Other findings, such as reduced waist circumference, obesity, glycated hemoglobin, and lipid levels, shared mixed results. At this time, we do not have a definition of what duration of device use is deemed standard for health. We have no consensus on which devices are superior health-wise. Our study, however, indicates that these devices, used with adequate health professional supervision, have a role to play in motivation and increased physical activity, enough to cause impactful gains in cardiovascular health.

## Introduction and background

Wearable electronic devices have gained popularity in recent years for their varied applications in communication and data collection, as well as their role in healthcare. In healthcare, uses range from step-counting, sleep monitoring, blood pressure, to pulse recording [1-3]. Recently, these devices have been reported to record arrhythmias and report remotely to health posts in case of emergencies [4]. Cardiovascular diseases are one of the leading causes of mortality worldwide [5]. In the United States, more than 650,000 deaths in 2020 were attributed to hypertension as a lead cause, according to the Centers for Disease Control and Prevention (CDC) [6]. Other comorbidities have been associated with poor cardiovascular outcomes, including diabetes, obesity, and hyperlipidemia [7]. Risk factors for developing cardiovascular disease may be classified as either modifiable or non-modifiable [8]. Modifiable risk factors describe what may be improved with intervention/behavioral change. Modifiable risk factors include smoking, level of physical activity, and diet. Non-modifiable risk factors include gender, age, and family history [8].

The last few years have seen several interventions with varying success levels in controlling the above modifiable risk factors. These include new exercise modalities, including yoga and tai-chi [9,10], the introduction of wearable electronic devices, including FitBits and iWatches [11], healthy eating options and diets, and the use of pedometers and calorie counters. While the popularity of these measures is rising, there are mixed reports regarding the long-term effects of these modalities on improving cardiovascular outcomes. Some of these interventions have been in place for less than a decade and may take a while to arrive at conclusive results.

In this systematic review, we wish to determine if, among the adult population aged >18 years, there has been documented demonstrable improvement in modifiable risk factors of heart disease among those exposed to emerging trends in wearable electronic devices (smartwatches, rings, pedometers). We wish to determine if there is an established trend of improvement in indices such as lipid levels, glycated hemoglobin, and body mass index (BMI) among those who rely on these devices. Moreover, we seek to establish if these devices are associated with increased early diagnosis of heart disease as predictive tools. Does using these devices decrease the risk of heart disease by a clinically notable amount?

## Review

Methods

This systemic review was conducted using the Preferred Reporting Items for Systemic Review and Meta-Analysis (PRISMA) 2020 guidelines [12].

Search Sources and Strategy

We searched PubMed, ScienceDirect, Research Gate, and Science.gov for relevant literature from the 26th to the 31st of December 2022. Our research question was to determine if wearable electronic devices had the impact of improving modifiable cardiovascular risk factors as a whole. We used search terms and keywords describing cardiovascular health, wearable electronic devices, and preventive medicine, such as primary prevention, prevention, smoking, sedentary lifestyle, diet, weight loss, exercise, physical activity, arrhythmias, heart disease, healthy eating, obesity, stress, psychological stress, diabetes, hypertension, fruits and vegetables, adults, smart watches, Fitbits, artificial intelligence, smart wearables, pedometer, BMI, weight loss, and wearable electronic devices in arriving at these records.

We removed one duplicate from the initial total of 63 articles. Subsequently, we assessed each article’s title, result, conclusion, and abstract for suitability. This allowed us to narrow down our numbers before applying our inclusion and exclusion criteria. After narrowing down this approach, we used standardized quality assessment tools based on the type of paper. We included 20 articles in this systematic review (Table 1). One article was later excluded as the authors retracted in the course of this review, leaving us with 19 articles.

**Table 1:** The databases used and the identified number of papers for each database. MeSH = Medical Subject Headings

Database used	Search strategy	Number of papers
PubMed	artificial intelligence AND cardiovascular outcomes AND blood pressure	30
ResearchGate	artificial intelligence AND cardiovascular outcomes AND blood pressure	8
Science.gov	artificial intelligence AND cardiovascular outcomes AND blood pressure AND smart watches AND BMI, 2017 - 2022	16
ScienceDirect	artificial intelligence AND cardiovascular outcomes AND blood pressure AND smart devices AND BMI AND primary prevention AND secondary prevention AND exercise AND smart watches	7
MeSH strategy PubMed	(“Wearable Electronic Devices/adverse effects”[Mesh] OR “Wearable Electronic Devices/classification”[Mesh] OR “Wearable Electronic Devices/economics”[Mesh] OR “Wearable Electronic Devices/ethics”[Mesh] OR “Wearable Electronic Devices/etiology”[Mesh] OR “Wearable Electronic Devices/history”[Mesh] OR “Wearable Electronic Devices/instrumentation”[Mesh] OR “Wearable Electronic Devices/methods”[Mesh] OR “Wearable Electronic Devices/microbiology”[Mesh] OR “Wearable Electronic Devices/nursing”[Mesh] OR “Wearable Electronic Devices/organization and administration”[Mesh] OR “Wearable Electronic Devices/parasitology”[Mesh] OR “Wearable Electronic Devices/pharmacology”[Mesh] OR “Wearable Electronic Devices/psychology”[Mesh] OR “Wearable Electronic Devices/standards”[Mesh] OR “Wearable Electronic Devices/statistics and numerical data”[Mesh] OR “Wearable Electronic Devices/supply and distribution”[Mesh] OR “Wearable Electronic Devices/therapeutic use”[Mesh] OR “Wearable Electronic Devices/therapy”[Mesh] OR “Wearable Electronic Devices/trends”[Mesh] OR “Wearable Electronic Devices/veterinary”[Mesh]) AND “Heart Disease Risk Factors”[Mesh]	1

Inclusion and Exclusion Criteria

We selected the latest literature and articles published in the past five years, between January 2017 and December 2022, including papers written in the English language or if the full-text English-language translation was available. We only included research papers involving the effects of wearable medical devices on humans. We excluded research papers if the full text could not be retrieved. Gray literature was also not included. Table 2 presents a summary of our inclusion and exclusion criteria.

**Table 2:** Inclusion and exclusion criteria.

Inclusion criteria	Exclusion criteria
Individuals, regardless of gender, greater than 18 years of age	Individuals less than 18 years of age
English-speaking population	Non-English-language literature
Use of wearable electronic devices for health promotion	Gray literature
Full-text literature published between 2017 and 2022	
Unspecified geographical location	

Selection Process

The population of interest was identified using keywords as identified above. In case of a conflict regarding eligibility, co-authors finalized the articles by mutual consensus. Microsoft Excel was employed in weeding out duplicates. Inclusion and exclusion criteria were applied to the shortlisted research papers, and only articles that satisfied the criteria were shortlisted.

Quality Assessment

All shortlisted articles were checked for quality using the relevant quality appraisal tools. A cut-off of 60% or above was used in the selection. The studies that satisfied the quality appraisal were included in the systematic review. In cases where tools could not decide, articles were selected based on suitability in addressing the research question posed (Table 3).

**Table 3:** Tools used for quality checks. JBI check tool = Joanna Briggs Institute check tool for evaluating case reports; AMSTAR = A Measurement Tool to Assess Systematic Reviews

Type of paper	Quality assessment tool
Case report	JBI check tool
Observational study	Newcastle-Ottawa
Systematic review	AMSTAR
Randomized control trial	Cochrane risk of bias

Data Collection Process

**Figure 1:**
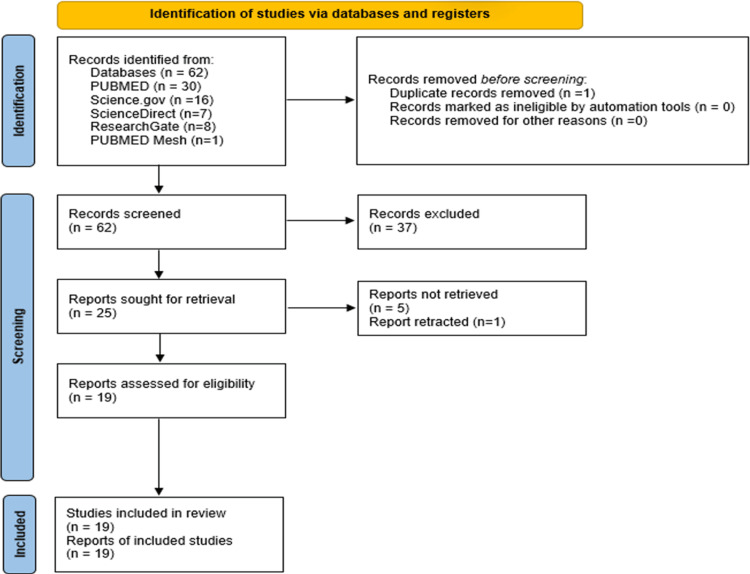
Preferred Reporting Items for Systematic Reviews and Meta-Analyses flow diagram.

After articles were selected, primary outcomes were assessed along with supporting information. Questionnaires were designed to organize the data into sets and units of argument for our review (Figure 1).

Results

A total of five databases were included in this study, including ResearchGate, PubMed, Science.gov, ScienceDirect, and the PubMed MeSH strategy. A total of 63 articles were initially selected and narrowed down to 20 based on the selection criteria outlined above. Four articles were systematic reviews and meta-analyses. Nine articles were observational studies. There was one case report and five randomized controlled trials. Most studies used wearable devices as the focus of their investigations [1,13-16], while others used these devices as part of a broader investigation [17-19]. Table 4 summarizes the study articles by type, quality assessment used, number of patients, study focus, and year of publication.

**Table 4:** Selected articles with years of publication. Articles are categorized into study types and quality assessment tools employed in evaluation. AMSTAR = A Measurement Tool to Assess systematic Reviews; JBI = Joanna Briggs Institute

Article type	Quality assessment tool	Research article	Year of publication	Number of patients	Objective/Focus of study	Conclusions
Systematic review	AMSTAR	Amaratunga et al. [1]	2020	1,973,129	Effect of machine learning in predicting clinical outcomes	Benefits in monitoring and predicting clinical parameters
		Dehghan et al. [13]	2022	2,373	Effect of wearable electronic device on the quality of life	Devices associated with improved physical activity
		Franssen et al. [14]	2020	8,147	Effect of wearable devices on health outcomes	Device use associated with improved blood pressure and waist circumference
		Jo et al. [15]	2019	1,615	Proof of wearable device benefits for heart disease	Low likelihood of benefit of device use on cardiovascular disease
Observational studies	Newcastle-Ottawa tool/JBI	OraLee et al. [16]	2022	864	Blood pressure and body weight changes with artificial intelligence-based coaching	Associated device use with decreased body weight
		Leopold et al. [2]	2021	307	Use of digital devices in evaluating the relationship between sleep and cardiovascular health	Three distinct sleep phenotypes identified with device use
		Mlakar et al. [17]	2018	24	Effect of telemonitoring on patient well-being	Useful in monitoring and predicting health outcomes and patient-reported feelings of health
		Cano et al. [4]	2022	69	Role of calibration in machine learning	Electronic monitoring helps distinguish hypertensive from normotensive patients
		Golbus et al. [11]	2021	6,765	Effect of wearable devices on healthcare	Potential for further studies in universalizing the data obtained for cardiovascular health
		Huh et al. [18]	2019	53	Effect of wearable devices on metabolic syndrome	Device use associated with increased physical activity and improved blood pressure
		Zompanti et al. [19]	2021	1	Evaluation of contactless wearable multi-lead EKG	Two or more lead EKG monitoring is not effective for ambulatory patients
		Johansson et al. [20]	2020	1,053	Differences in high versus low-intensity exercise monitored by wearable devices	Older adults benefit most from low-intensity exercise while adults in general benefit from high intensity
		Rykov et al. [3]	2020	83	Association between wearable devices and modifiable biomarkers of cardiometabolic disease	Wearable devices provide accurate and meaningful information which may predict the risk of cardiovascular disease
Randomized controlled trials	Cochrane risk of bias tool	O'Brien et al. [21]	2020	53	Activity trackers and feedback systems used in kidney transplant recipients	Increased daily steps noted in the intervention group
		Roberts et al [22]	2019	40	Effects of wearable devices on daily activity	Overall, positive influence of devices on daily activity
		Christen et al. [23]	2020	62	Impact of dark chocolate on blood pressure as measured by mobile health technology	Short-term dark chocolate use did not lower blood pressure
		Coffeng et al. [24]	2017	850	Device-monitored lifestyle intervention at worksites	Significant improvement in cardiovascular and health metrics
		Broers et al. [25]	2020	34	Effects of lifestyle intervention on data derived from wearable devices	Better lifestyle behaviors with the introduced intervention
Case report	JBI check tool	Narita et al. [26]	2020	1	Effects of ICT-based multisensory ambulatory blood pressure monitoring	Actisensitivity devices detected cardiac systolic function more sensitively than conventional methods

We identified a total of 15 systematic reviews using the search criteria outlined above. In assessing the methodological quality of systematic reviews tool, we used a cut-off of 60%, with four articles satisfying our initial preset quality goal, and 11 articles were rejected (Table 5).

**Table 5:** AMSTAR quality assessment tool. AMSTAR = A measurement tool to assess systematic reviews. Key: + = Yes; - = No; ? = Uncertain. Q1 - Did the research questions and inclusion criteria for the review include the components of PICO? Q2 - Did the report of the review contain an explicit statement that the review methods were established prior to the conduct of the review, and did the report justify any significant deviations from the protocol? Q3 - Did the review authors explain their selection of the study designs for inclusion in the review? Q4 - Did the review authors use a comprehensive literature search strategy? Q5 - Did the review authors perform study selection in duplicate? Q6 - Did the review authors perform data extraction in duplicate? Q7 - Did the review authors provide a list of excluded studies and justify the exclusions? Q8 - Did the review authors describe the included studies in adequate detail? Q9 - Did the review authors use a satisfactory technique for assessing the risk of bias (RoB) in individual studies that were included in the review? Q10 - Did the review authors report on the sources of funding for the studies included in the review? Q11 - Did the review authors use appropriate methods for the statistical combination of results, if a meta-analysis was performed? Q12 - Did the review authors assess the potential impact of RoB in individual studies on the results of the meta-analysis or other evidence synthesis? Q13 - Did the review authors account for RoB in individual studies when interpreting/ discussing the results of the review? Q14 - Did the review authors provide a satisfactory explanation for, and discussion of, any heterogeneity observed in the results of the review? Q15 - Did the review authors carry out an adequate investigation of publication bias (small study bias) and discuss its likely impact on the results? Q16 - Did the review authors report any potential sources of conflict of interest, including any funding they received for conducting the review?

AMSTAR quality assessment tool	Q1	Q2	Q3	Q4	Q5	Q6	Q7	Q8	Q9	Q10	Q11	Q12	Q13	Q14	Q15	Q16
Amaratunga et al. [1]	-	-	+	+	+	-	-	+	+	+	+	+	+	+	-	+
Dehghan et al. [13]	+	+	-	-	-	-	-	+	+	+	+	+	-	+	-	+
Franssen et al. [14]	+	+	+	+	+	?	?	+	+	?	+	+	+	+	?	+
Jo et al. [15]	+	+	+	+	?	+	+	+	+	+	-	-	+	?	?	+

We identified 20 observational studies using the selection criteria outlined above. We rejected 11 of these articles and accepted nine based on a preset quality goal of more than 60% rating for each subcategory using the Newcastle-Ottawa Tool (Table 6).

**Table 6:** Newcastle-Ottawa tool. *: Number correlates with assessment findings for each category.

	Selection	Comparability	The outcome of interest was not present at the start of the study
OraLee et al. [16]	****	*	***
Leopold et al. [2]	****	*	*
Mlakar et al. [17]	****	**	**
Cano et al. [4]	****	*	**
Golbus et al. [11]	****	*	***
Huh et al. [18]	**	**	**
Zompanti et al. [19]	****	**	*
Johansson et al. [20]	****	**	**
Rykov et al. [3]	**	*	*

We identified a total of 23 randomized control trials following the selection process described above. We accepted five articles for the purposes of this study and rejected 18 for qualitative reasons (Table 7).

**Table 7:** Cochrane risk of bias tool. Key: + = Yes; - = No; ? = Uncertain.

Studies included	Random sequence generation (selection bias)	Allocation concealment (selection bias)	Blinding of participants and personnel	Blinding of the outcome of assessment (detection bias)	Judge risk of bias for each domain	Selective reporting (reporting bias)	Integrate judgment into results and conclusions
O'Brien et al. [21]	+	+	+	+	-	+	+
Roberts et al. [22]	+	+	-	-	+	+	+
Christen et al. [23]	-	+	-	-	+	+	+
Coffeng et al. [24]	+	-	+	+	+	+	+
Broers et al. [25]	+	?	+	+	+	+	+

Using our selection criteria, we identified four case reports which met our initial search parameters. Only one was selected based on our quality checks, using the JBI check tool (Table 8).

**Table 8:** JBI check tool. JBI = Joanna Briggs Institute. Key: + = Yes; - = No; ? = Uncertain.

	Were the patient’s demographic characteristics clearly described?	Was the patient’s history clearly described and presented as a timeline?	Was the current clinical condition of the patient on presentation clearly described?	Were diagnostic tests or assessment methods and the results clearly described?	Was the intervention(s) or treatment procedure(s) clearly described?	Was the post-intervention clinical condition clearly described?	Were adverse events (harms) or anticipated events identified and described?	Does the case report provide takeaway lessons?
Narita et al. [26]	+	-	+	+	+	+	-	+

Table 9 presents the areas of emphasis of each article under review, as was highlighted in the ensuing discussion.

**Table 9:** Areas of emphasis of each study. + = addressed by the study. A blank indicates that the associated category was not addressed in the study.

Research article	Year of publication	Current perspectives on artificial intelligence in healthcare vs. in the past	Evidence for conflicting findings on a wearable device as a regular tool	Papers addressing the research question	Future of wearable electronic devices
Amaratunga et al. [1]	2020	+		+	+
Dehghan et al. [13]	2022	+		+	
Franssen et al. [14]	2020	+		+	
Jo et al. [15]	2019	+	+	+	
OraLee et al. [16]	2022	+	+	+	+
Leopold et al. [2]	2021	+			+
Mlakar et al. [17]	2018	+	+		
Cano et al. [4]	2022		+		+
Golbus et al. [11]	2021	+	+		
Huh et al. [18]	2019	+	+	+	
Zompanti et al. [19]	2021	+			+
Johansson et al. [20]	2020		+		+
Rykov et al. [3]	2020	+			+
O'Brien et al. [21]	2020	+	+		
Roberts et al. [22]	2019	+			
Christen et al. [23]	2020		+	+	+
Coffeng et al. [24]	2017		+		+
Broers et al. [25]	2020		+		
Narita et al. [26]	2020	+	+		

Discussion

Perspectives on the Use of Wearable Electronic Devices in Healthcare

Wearable electronic devices have progressively gained popularity in recent years for their reported ability to improve physical activity [2]. They have also been used to track changes in sleep [2]. These devices have also been used to gather large datasets for qualitative and quantitative evaluation of various health indices, including developing prediction models for adverse cardiac outcomes [1,3]. Attempts have been made to establish the link between these devices and quality of life [13], and, increasingly, they have been linked with increased ease of access to health professionals, highlighting their use as communication devices [13]. Attempts have also been made to link these devices with improved outcomes concerning cardiovascular disease [13,14]. Some data have connected these devices with improvement in metabolic syndrome [18], citing their role in behavioral changes/fitness and blood pressure.

While changes in physical activity levels have been well documented as an incontrovertible benefit of using these devices, it can be argued that they do not achieve this in isolation [13-15]. Other interventions must be introduced to augment the role of these instruments or realize their full potential [13-15]. There is no doubt that added supervision of patients is associated with better cardiovascular outcomes rather than self-monitoring alone [16]. Therefore, in instances of professional mentorship toward better cardiac health, while monitoring devices are helpful, they may not necessarily be central and cannot be regarded as a game-changer [16]. Due to the effect on physical activity, sedentary lifestyles, including occupations that do not demand physical intensity, appear to stand more to gain from these devices [3,22,27]. The question then becomes, “what is the main role of these devices on modifiable cardiovascular disease risk factors other than increased physical activity?.”

Increased motivation for physical activity is commendable, however, in keeping with the more helpful aim of health-related softer endpoints, including quality of life assessments, as opposed to harder endpoints such as mortality, even in previously diagnosed individuals [17]. To improve this observed effect, clear parameters have to be set as to what is standard device use and what is not. Wearable devices have been used to document differences in, among other parameters, heart rate and blood pressure in various ethnic and gender groups. The impact of this technology has also been investigated by demographics [11], which introduces a new category for classification/standardization. Still, the singular relevance of these findings is yet to be established clinically [11]. However, the use of these devices continues to evolve. For instance, studies are ongoing for non-contact ambulatory electrocardiogram (EKG) monitoring [19]. Other findings support using these devices for risk prediction in cardiovascular outcomes [1,3].

Beyond recording, documenting, and predicting outcomes, these devices have found their way into actual clinical practice. Aside from primary prevention of heart disease in various age categories [1,22], secondary outcomes have been studied for renal and previously diagnosed heart disease, including hypertension [16,18,21,25], with improved physical activity being noted as the main benefit.

Conflicting Data on the Universalization of Wearable Electronic Devices

Increased physical activity is likely the most profound benefit of wearable devices [3,13-16,18,22,25,27]. Physical activity is a modifiable risk factor [8]. There are other behavioral components impacting cardiovascular health, including sleep, diet, and smoking cessation [8]. While there is evidence that devices are developing in these areas [6,28,29], electronic devices are yet to catch up in utility in these areas as they have for physical activity.

The devices alone, in some studies, have not been shown to improve cardiovascular outcomes [16], requiring additional mentoring to gain the full anticipated effects. Other studies have shown no benefits outside of physical activity motivation and increase [15]. Furthermore, the varied forms of physical activity per age group require a degree of individualization of these devices [11]. While some studies note an increase in physical activity with improvement in metabolic syndrome [3,22,27], they fail to give a clear individualized set-point at which device use, by the duration of wearing, or by other measures may be deemed optimal. The idea of adherence to these devices is undefined [14,18] and cannot be delineated from the intense supervision mentioned in the studies. There is no single definition of adherence.

The potential for data acquisition for various purposes, even outside healthcare, is undoubted [1,3,11]. Wearable devices provide ample data of varied or even questionable utility [4,11], including monitoring heart failure patients [4,17]. However, there appears to be no universalization or standardization of findings from these devices to take advantage of this data density [1,4,17]. There remains work to be done in this area in incorporating the diversity and abundance of data into mainstream medicine [11].

One of the articles within a systematic review pointed to the positive effect of using a wearable device on glycated hemoglobin in older adults [15]. Unfortunately, this finding was not replicated in the same study across other articles, decreasing confidence in this review alone as the basis for recommending wearable electronic devices among all age groups. Using data mining, it has also been suggested that telemonitoring may be used to predict the subjective feeling of health [17]. Subsequent data, however, seem to suggest the contrary [11]. The difference seems to lie in the sophistication of measurement tools used in the former study compared to the watch used in the latter, which is sponsored [11]. If the use of these devices is to be promoted among diverse ethnic groups [11,18], simplicity must be factored into its use. Finally, the evidence for the utility of these devices seems to tilt in favor of those with sedentary lifestyles [22,24,27]. There appears to be no additional benefit to acquiring these devices for people who already engage in healthy physical activity beyond better data-gathering and record-keeping [11].

Do Wearable Electronic Devices Impact the Causes of Modifiable Risk Factors Beyond Physical Activity?

Modifiable risk factors contributing to cardiovascular health are impacted by behavior. They include smoking cessation, physical activity, healthy eating habits, obesity, high blood pressure, and diabetes [30]. In the majority of records reviewed in this article, physical activity was noted to be the foremost direct beneficiary of the use of wearable electronic devices [3,11,13-16,18,21-24,27]. According to the CDC, adults need at least 150 minutes of moderate physical activity per week, or 75-150 minutes weekly of vigorous-intensity aerobic physical activity, to be considered to have undergone adequate health promotion [31]. The CDC also notes that some activity is better than none. Therefore, overall, the idea of increasing physical activity alone is laudable. Physical activity is linked to improved insulin sensitivity, cardiovascular disease risk, and metabolic syndrome [32]. Whether this improvement requires additional supervision remains debatable, with the consensus tilting toward the affirmative. Most sources stress the role of additional supervision/consultation/coaching [14,16,20,21], emphasize a physician-led role in using these devices [1,3], or the need for the link with established practice for professional inputs [3,13,25,28].

Aside from the direct role of wearable electronic devices on physical health, findings for other facets of health have been mixed. According to Ara et al. [15], in a study that evaluated hypertension, diabetes, cholesterol, and weight loss, these devices do not offer consistent health benefits outside of increased motivation for physical activity. This assertion has been supported by other studies [22], which, in addition to the above, note no improvement in weight or sleep quality with electronic supervision. However, improvements were noted in systolic blood pressure, waist circumference, weight loss, cholesterol, and diabetes control in other studies [14,15].

The future of wearable devices appears promising. Research in this area shows promise with more accurate, age-sensitive blood pressure measurements [1,16,20], and better control in those with metabolic syndrome using these devices [18]. Research also promises wearable devices that monitor EKGs beyond one lead offered by watches [19]. The new devices are increasingly targeted toward predicting cardiovascular outcomes [1,3,11,17]. Furthermore, wearable devices have helped in debunking some myths [23]. For instance, the effect of dark chocolate as having antihypertensive properties was debunked with the aid of these tools [23]. Electronic devices promise to do more by predicting cardiovascular outcomes [23,24] ahead of conventional methods in the future.

Limitations of the study

All data chosen were from developed countries. Resource-poor or developing countries have not seen similar studies, though this was not an exclusion criterion. This places wearable devices among what may be deemed a resource-rich health resource. Second, the studies failed to disclose the full financial costs of adherence to programs and the generalizability of findings among the less-resourced areas. This would have been helpful in gaining insights into the motivations or the lack thereof in procuring the devices. Third, there is no standardization in the way data are collected between devices from different companies and possibly different devices of the same company. To gain entry into mainstream healthcare, this must be addressed down the line. Fourth, other modifiable risk factors, such as smoking and healthy eating, need to be further investigated. Fifth, portions of the data were sponsored by reasonably biased parties. Tech companies are likely to endorse the positive health benefits of their products. Sixth, despite the degree of promise, wearable devices continue to be limited by adherence, availability of electricity, and the internet. Finally, standard device use remains undefined. We have not even begun to draw the line between device addiction and appropriate use.

## Conclusions

We assessed the influence of wearable electronic devices on cardiovascular disease. Our aim was to determine the empirical role of these devices on heart disease. The effect of these devices has long been recognized and marketed by various companies as health-promoting. They continue to gain popularity, although the exact definition of what aspect of health they promote remains poorly defined. Regardless of this limitation, emerging technologies continue to be developed, promising real-time monitoring and prediction tools as an adjunct to diagnosing and treating heart disease. This research is important because, increasingly, patients continue to procure various forms of wearable devices to improve their health and often ask for options, opinions, and affirmation from their physicians.

While this is commendable, our evaluation of the devices shows them to be mainly motivational tools promoting physical activity. The extent of this physical activity cannot be entirely influenced by the devices alone, requiring additional guidance, in many instances, from healthcare providers for optimal health outcomes. Outside physical activity increases, these devices have not shown consistent improvements in other indices of health. The impact and future of these devices on other indices of health (smoking, diabetes, obesity) are uncertain at this time. More studies are needed to explore device use definitively and conclusively, assess the role of wearable electronic devices as significant agents of improved cardiovascular health, or recommend them formally in the primary care setting.
