# Correction: Exploring the Role of Wearable Electronic Medical Devices in Improving Cardiovascular Risk Factors and Outcomes Among Adults: A Systematic Review

**DOI:** 10.7759/cureus.c312

**Published:** 2025-09-23

**Authors:** Kofi Seffah, Mustafa Abrar Zaman, Nimra Awais, Travis Satnarine, Ayesha Haq, Grethel N Hernandez, Safeera Khan

**Affiliations:** 1 Internal Medicine, California Institute of Behavioral Neurosciences & Psychology, Fairfield, USA; 2 Internal Medicine, Piedmont Athens Regional Medical Center, Athens, USA; 3 Research, California Institute of Behavioral Neurosciences & Psychology, Fairfield, USA; 4 Pediatrics, California Institute of Behavioral Neurosciences & Psychology, Fairfield, USA

This article has been corrected to fix the following typo in the PRISMA diagram: "Records identified" corrected from 63 to 62.

